# Verotoxin-1-Induced ER Stress Triggers Apoptotic or Survival Pathways in Burkitt Lymphoma Cells

**DOI:** 10.3390/toxins12050316

**Published:** 2020-05-11

**Authors:** Justine Debernardi, Catherine Pioche-Durieu, Eric Le Cam, Joëlle Wiels, Aude Robert

**Affiliations:** 1 UMR 8126 CNRS, Institut Gustave Roussy, Université Paris-Saclay, 94805 Villejuif, France; justine.debernardi@gmail.com; 2 UMR 7592 CNRS, Institut Jacques Monod, Université Paris Diderot-Paris 7, 75205 Paris CEDEX 13, France; Catherine.DURIEU@ijm.fr; 3 UMR 8200 CNRS, Institut Gustave Roussy, Université Paris-Saclay, 94805 Villejuif, France; eric.lecam@gustaveroussy.fr; 4 INSERM U1279, Institut Gustave Roussy, Université Paris-Saclay, 94805 Villejuif, France

**Keywords:** shiga toxins, Gb3/CD77, apoptosis, ER stress, autophagy, Burkitt lymphoma

## Abstract

Shiga toxins (Stxs) expressed by the enterohaemorrhagic *Escherichia coli* and enteric *Shigella dysenteriae 1* pathogens are protein synthesis inhibitors. Stxs have been shown to induce apoptosis via the activation of extrinsic and intrinsic pathways in many cell types (epithelial, endothelial, and B cells) but the link between the protein synthesis inhibition and caspase activation is still unclear. Endoplasmic reticulum (ER) stress induced by the inhibition of protein synthesis may be this missing link. Here, we show that the treatment of Burkitt lymphoma (BL) cells with verotoxin-1 (VT-1 or Stx1) consistently induced the ER stress response by activation of IRE1 and ATF6—two ER stress sensors—followed by increased expression of the transcription factor C/REB homologous protein (CHOP). However, our data suggest that, although ER stress is systematically induced by VT-1 in BL cells, its role in cell death appears to be cell specific and can be the opposite: ER stress may enhance VT-1-induced apoptosis through CHOP or play a protective role through ER-phagy, depending on the cell line. Several engineered Stxs are currently under investigation as potential anti-cancer agents. Our results suggest that a better understanding of the signaling pathways induced by Stxs is needed before using them in the clinic.

## 1. Introduction

Shiga toxins (Stxs), also known as verotoxins (VTs) or Shiga-like toxins (SLTs), are a family of cytotoxic proteins, structurally and functionally related, that are produced by the enteric pathogens *Shigella dysenteriae* type 1and Stx-producing *Escherichia coli* (STEC). Two major types of Stxs have been described, VT-1 (or Stx1) and VT-2 (or Stx2), which display 56% amino-acid identity. A broad spectrum of human diseases is associated with Stx-producing organisms, ranging from mild watery diarrhea to bloody diarrhea, hemorrhagic colitis, and life threatening hemolytic uremic syndrome (HUS). Infection with Stx-producing bacteria continues to be a significant worldwide public health problem. In the absence of a vaccine or effective therapy to treat the disease, prevention and supportive therapies are currently the main tools to fight such contamination [1,2]. An improved understanding of host-cell responses to Stxs would allow the development of more effective treatment. In addition, the identification of intermediate signaling molecules in Stx-induced pathways may constitute therapeutic targets to limit the tissue damage caused by Stxs.

Members of the Stx family consist of a single 32-kDa A-subunit in non-covalent association with five B-subunits. The B-subunit pentamers form a doughnut-shaped structure that recognizes the cell surface receptor. For nearly all Stxs, this receptor is the neutral glycosphingolipid globotriaosylceramide (Gb3) but Stx2e (responsible of the porcine edema disease) preferentially binds to globotetraosylceramide (Gb4) [3,4]. Following Gb3 binding, Stxs are internalized and undergo retrograde transport through the Golgi to the lumen of the endoplasmic reticulum (ER) [5]. In the ER, the A-subunits are proteolytically cleaved into 27 kDa fragments that translocate to the cytoplasm. This active A-subunit is an N-glycosidase which inhibits protein synthesis by removing an adenine from 28S RNA [6]. 

Deregulation of Gb3 expression has been observed in various malignancies. Gb3 is highly expressed in Burkitt lymphoma (BL) cells [7] and in diverse types of solid tumors, including breast, testicular, and ovarian carcinomas [8,9,10]. Interestingly, a new imaging technology based on mass spectrometry (MALDI-2-MSI) has been recently developed to study the precise localization of Gb3 containing various fatty acid moieties and of its precursors which should improve our understanding of glycosphingolipid metabolism in cancer cells [11]. The concept of using Stx and its non-active binding subunit, StxB (as a delivery tool), for therapy emerged from cell trafficking experiments performed in the 1990s. Various preclinical studies have been conducted with this toxin. Regression of the tumor mass has been observed in various xenograft models, but the strong cytotoxicity (protein synthesis arrest and induction of apoptosis) of VT-1 can cause significant side effects, especially in normal cells expressing Gb3. Attempts have thus been made to reduce the doses and/or use modified versions of the toxin [12]. 

Although the cytotoxic pathway induced by these toxins may differ slightly between diverse cell types, it is now clear that they induce cell death through apoptosis. The apoptotic process generally depends on both caspases and molecules stored in mitochondria [13,14,15] but there are a few exceptions like HeLa cells where the process is mitochondria-independent [16]. We have further explored the signal transduction pathway induced by VT-1 in BL cells and showed that it is a relatively conventional caspase- and mitochondria-dependent pathway, except for the role of BID (a proapoptotic member of the BCL-2 family), since both the full-length and truncated forms of this protein are involved in the process [17,18,19]. Others have shown that the ER stress response induced by Stxs/VTs in monocytic THP1 cells contributes to caspase 8 activation and thus also takes part in the apoptotic pathway. In these cells, the B-subunit or the holotoxin containing a mutation-induced inactivated A subunit does not induce apoptosis [13]. These data suggest that the delivery of functional holotoxins to the ER is needed to induce apoptosis.

The ER is an organelle with essential functions in eukaryotic cells. It is both the primary site for the correct folding and processing of proteins for secretion or insertion into the cellular membrane and a major intracellular calcium store. The status of protein folding and Ca^2+^ storage is controlled by three major ER stress sensors: the protein IRE1 (inositol requiring enzyme 1), the serine/threonine kinase PERK (PKR-like ER protein kinase), and the transcription factor ATF6 (activating transcription factor 6). These proteins are associated with the chaperone BIP (binding immunoglobulin protein, also called GRP78 or HSPA5). When unfolded proteins accumulate in the ER, BIP dissociates from the sensors, thus allowing their activation [20]. PERK and IRE1 are activated by homo-dimerization and autophosphorylation, whereas ATF6 activation requires translocation to the Golgi and proteolytic cleavage. ER membrane sensors activate signaling pathways that result in transient attenuation of overall protein translation and in activation of the transcription of genes encoding proteins involved in the degradation of misfolded proteins via the ER associated protein degradation pathway (ERAD). This coordinated response is called the unfolded protein response (UPR). Failure to correct protein folding defects or maintain Ca^2+^ homeostasis induces prolonged signaling through the UPR, leading to apoptosis. C/REB homologous protein (CHOP, also known as GADD153) is a key transcription factor involved in UPR which, directly or indirectly, regulates the expression of genes involved in apoptosis, [21,22,23,24]. How the UPR switches from the pro-survival to pro-death mode still remains unclear.

In this work, we aimed to better delineate the early stages of the VT-1-induced apoptotic pathway in BL cells to clarify the involvement of ER stress. 

## 2. Results

### 2.1. ER-Phagy Can Alter VT-1-Induced Apoptosis

**Figure 1:**
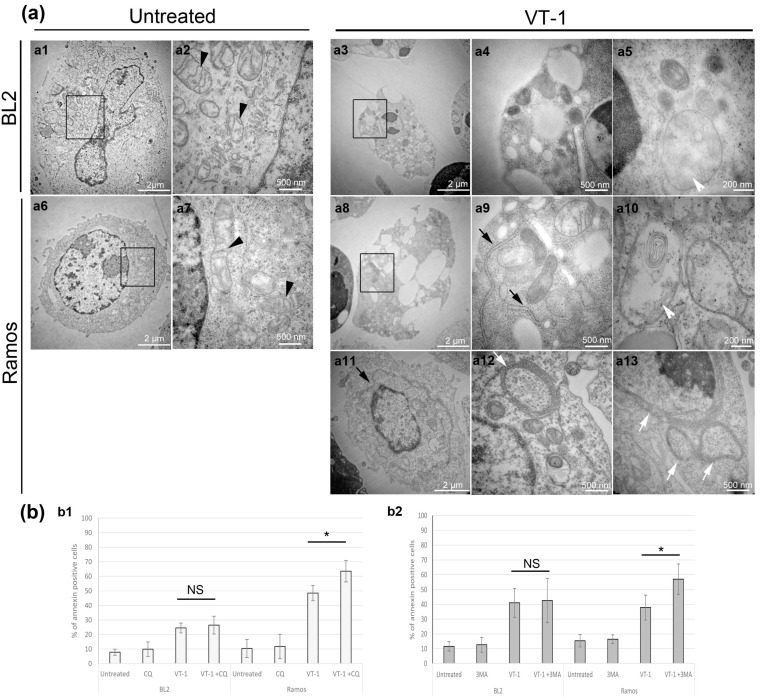
Verotoxin-1 (VT-1) induced protective endoplasmic reticulum (ER)-phagy in certain Burkitt lymphoma (BL) cell lines. (**a**) Effect of VT-1 treatment observed by electron microscopy. (**a1**–**a5**) BL2 cells; (**a6**–**a13**) Ramos cells. (**a2**,**a4**,**a7**,**a9**) show magnifications of (**a1**,**a3**,**a6**,**a8**), respectively. Black arrow head: mitochondria with lamellar cristae, white arrow head: mitochondria with the loss of cristae and empty spaces, black arrow: expanded peripheral ER, cytoplasmic ER extension; white arrows: ring-shaped ER whorls. Cells in (**a3**–**a5**) and (**a8**–**a10**) were treated with VT-1 for 6 h and for 4 h in (**a11**–**a13**). (**b**) Effect of chloroquine (CQ) and 3-methyladenine (3MA), two different autophagy inhibitors on VT-1-induced apoptosis. Cells were pretreated with CQ (**b1**) or 3MA (**b2**) for 30 min and subsequently with VT-1 for 6 h. Cells were then analyzed by flow cytometry after annexin V/PI labeling. Statistical test: Mann Whitney, * *p* < 0.05. NS, non significant. Scale bars (**a1**,**a3**,**a6**,**a8**,**a11**), 2 μm; (**a2**,**a4**,**a7**,**a9**,**a12**,**a13**), 500 nm; (**a5**,**a10**), 200 nm.

We first analyzed by electronic microscopy (EM) the ultrastructure of two different BL cell lines (BL2 and Ramos) treated or not with VT-1. Before treatment, mitochondria of various sizes displayed typical ultrastructure, with the inner membrane projecting into the matrix at crista junctions to form lamellar cristae (BL2, Figure 1(a1,a2); Ramos, Figure 1(a6,a7), black arrowhead). After 6 h of treatment with VT-1, apoptosis was clearly induced in both cell lines; most cells had a fragmented nucleus and a dark cytoplasm containing vacuoles. Furthermore, we noticed marked changes in the mitochondrial ultrastructure; some mitochondria became darker and smaller, suggesting that they were fragmented, and some showed the loss of cristae, with empty spaces (BL2, Figure 1(a3–a5); Ramos, Figure 1(a8–a10), white arrowhead). This aspect of the mitochondria is consistent with membrane permeability and apoptogen factor release.

However, we also observed additional features in Ramos, reminiscent of the morphology of autophagic organelles. At an earlier timepoint of treatment (4 h) and in some cells at 6 h, we observed large vesicles that resembled autophagosomes, except that the delimiting outer membranes were densely studded with ribosomes, suggesting that the membranes were derived from the ER (Figure 1(a12,a13), white arrows, named ring-shaped ER whorls). We also observed an expanded and more peripheral rough ER (Figure 1(a11), black arrow) and more cytoplasmic rough ER extensions (Figure 1(a9), black arrows), which are characteristics of ER-phagy.

We thus evaluated the effects of autophagy inhibitors such as chloroquine (CQ, Figure 1(b1)) and 3-methyladenine (3MA, Figure 1(b2)) on VT-1-induced apoptosis. Neither CQ nor 3MA had any effects on the induction of apoptosis in BL2 cells (26 ± 6% of apoptotic cells with VT-1 and CQ versus 25 ± 3% with VT-1 alone and 41 ± 10% with VT-1 and 3MA versus 43 ± 15% with VT-1 alone). By contrast, treatment of Ramos cells with autophagy inhibitors increased VT-1-induced cytotoxicity (from 49 ± 5% to 64 ± 7% for CQ and from 38 ± 8% to 57 ± 10% for 3MA), suggesting that autophagy, and more precisely ER-phagy, protects these cells from VT-1 induced cell death.

### 2.2. VT-1 Induces the ER Stress Response in Burkitt lymphoma Cells Though Activation of the Sensors ATF6 and IRE1

Common upstream signaling pathways are involved in ER stress-induced apoptosis and autophagy. We thus tested the activation of three key molecules of these pathways: ATF6, PERK, and IRE1.

**Figure 2:**
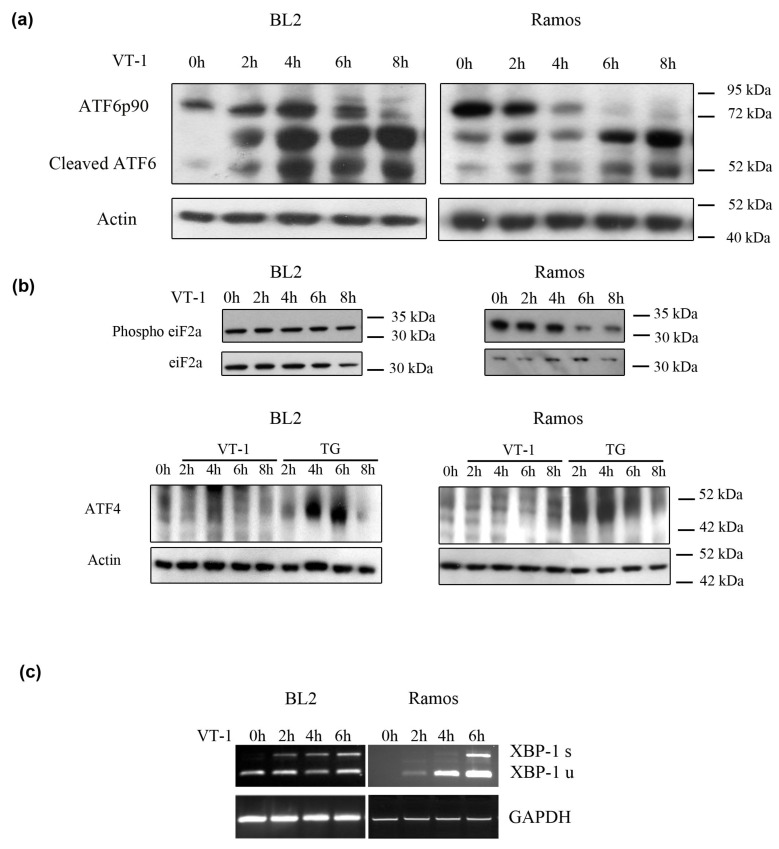
VT-1 activated ER stress sensors in BL cells. BL2 and Ramos cells were stimulated with VT-1 for 0, 2, 4, 6, or 8 h. (**a)** After treatment, cell lysates were prepared and ATF6 cleavage analyzed by western blotting. (**b**) Phospho-eIF2α, eIF2α, and ATF4 expression were also analyzed by western blotting. (**c**) After treatment, total RNA was isolated and amplified using XBP-1 specific primers. Amplification products were then incubated with the enzyme ApaLI. Unspliced XBP-1 (XBP-1 u cleaved by ApaLI into stackable ~280 and ~340 bp fragments) and spliced XBP-1 (XBP-1 s, 590 Bp, insensitive to ApaLI) were detected on agarose gels.

During ER stress, the inactive 90-kDa ATF6 protein undergoes proteolysis, leading to the release of a 50-kDa protein with transcriptional activity [25]. Treatment of BL cells with VT-1 resulted in the progressive cleavage of the inactive 90-kDa ATF6 into the active 50-kDa form (cleaved ATF6), with the complete disappearance of the full-length form after 8 h (Figure 2a). However, the kinetic of ATF6 activation was faster in Ramos than BL2 cells. 

Activated PERK can phosphorylate the α subunits of eukaryotic initiation factor 2 (eIF2), a major regulator of mRNA translation [26]. We, therefore, measured the presence of phosphorylated eiF2α before and after VT-1 treatment by western-blot analysis (Figure 2b). There were no changes in eiF2α phosphorylation levels in BL2 cells, even after 8 h of treatment. On the contrary and intriguingly, the levels of phospho-eiF2α in Ramos cells decreased, beginning after 6 h of VT-1 treatment. The activation of PERK signaling also results in the attenuation of overall protein translation, concomitant with the induction of translation of only selective mRNA molecules, including those of activating transcription factor 4 (ATF4). We therefore also analyzed ATF4 expression after treatment of our BL cell lines with VT-1 (Figure 2b). We did not observe any increases in ATF4 levels, neither in BL2 nor Ramos cells, whereas ATF4 was clearly induced in these cells when they were treated with Thapsigargin, a well-known stress inducer. These results show that the PERK/eIF2α/ATF4 signaling pathway was not activated by VT-1 treatment of BL cells.

We then investigated activation of the IRE1 signaling pathway. IRE1 activation triggers splicing of the RNA transcript encoding the transcription factor XBP-1 [27]. One consequence of such splicing is the removal of an ApaLI site in the XBP-1 transcript. Thus, when incubated with ApaLI, the spliced XBP-1 transcript (XBP-1s) remains ~590 bp, whereas the unspliced XBP-1 transcript (XBP-1 u) is cleaved into ~280 and ~340-bp fragments (which are indistinguishable on gels). We thus extracted total mRNA of BL cells treated or not with VT-1 and amplified it using XBP-1 specific primers. The amplification products were then incubated with the enzyme ApaLI. XBP-1s was not present in untreated cells but gradually increased after treatment of both BL cell lines with VT-1, indicating IRE1 activation (Figure 2c). Contrary to ATF6, IRE1 appeared to be activated more quickly in BL2 than Ramos cells. 

**Figure 3:**
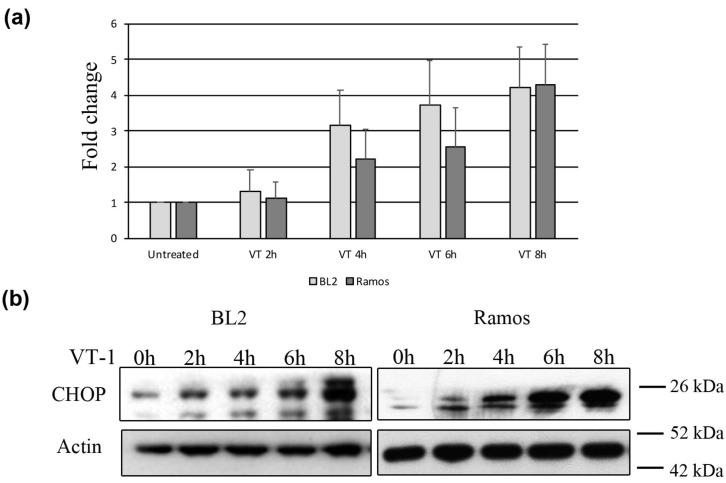
C/REB homologous protein (CHOP) expression increases in BL cells treated with VT-1. BL2 and Ramos cells were treated with VT-1 for 0, 2, 4, 6, or 8 h. After treatment, total RNA or protein extracts were prepared and CHOP induction analyzed by qRT-PCR (**a**) or western blotting (**b**).

One of the hallmarks of ER stress-sensor activation is increased expression of the transcription factor CHOP. Thus, we analyzed CHOP expression at various times after VT-1 treatment both by qRT-PCR and western blotting. CHOP was constitutively expressed at low levels in non-treated cells and gradually increased after VT-1 treatment, both at the RNA (Figure 3a) and protein levels (Figure 3b).

Overall, our results show that VT-1 induces an ER stress response in both BL cell lines but only at the transcriptional regulation level (through ATF6 and IRE1) and not at the translational level (PERK/eIF2α/ATF4).

### 2.3. Silencing CHOP Protects BL2 but Not Ramos Cells from VT-1 Induced Apoptosis

**Figure 4:**
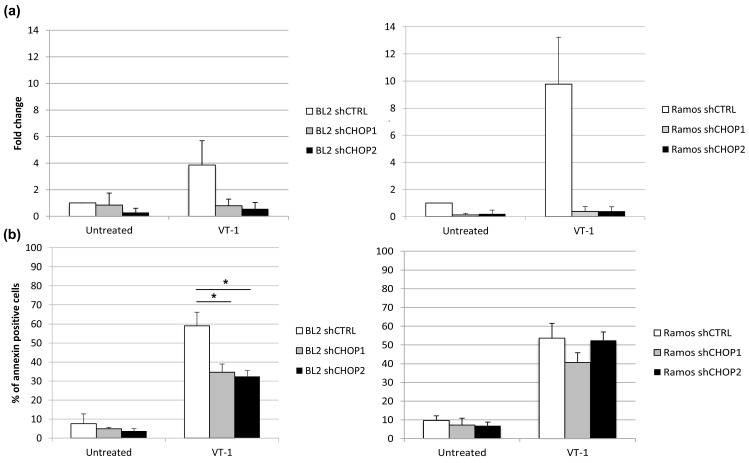
CHOP knockdown reduces VT-1-induced apoptosis in BL2 but not Ramos cells. After incubation of the cells with VT-1 for 6 h, the induction of CHOP was quantified by qRT-PCR (**a**) and the percentage of apoptotic cells analyzed by flow cytometry using annexinV/propidium iodide staining (**b**). Data are presented as the means ±SD from at least three independent experiments. Statistical test: Mann Whitney, * *p* < 0.05.

Given the results to this point, we tested the involvement of CHOP in VT-1 induced apoptosis by inhibiting its expression. A lentiviral vector-based shRNA system was used to stably repress CHOP expression in Ramos and BL2 cells. Cells obtained with two different shRNA constructs or a control shRNA were treated with VT-1 and the CHOP mRNA level determined by qRT-PCR to verify the knockdown of CHOP. The induction of CHOP was clearly observed after VT-1 treatment of shCTRL cells but not BL2 shCHOP or Ramos shCHOP cells (Figure 4a). We then assessed apoptosis by flow cytometry. CHOP knockdown did not reduce VT-1-induced apoptosis in Ramos cells (53.6 ± 8%, 40.7 ± 5%, and 52.5 ± 4% apoptosis after 6 h of VT-1 treatment of Ramos shCTRL, Ramos shCHOP1, and Ramos shCHOP2, respectively) but protected BL2 cells, since the percentage of apoptosis was reduced by almost two times (59 ± 7%, 34.6 ± 4%, and 32.5 ±3% apoptosis after 6 h of VT-1 treatment of BL2 shCTRL, BL2 shCHOP1, and BL2 shCHOP2, respectively, Figure 4b). 

**Figure 5:**
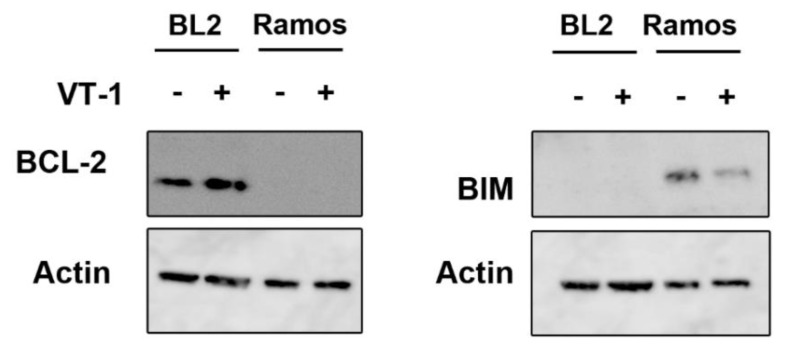
BCL-2 and BIM expression in BL cell lines by western blotting. Cells were incubated or not with VT-1 for 6 h.

Previous reports have shown CHOP to be involved in ER stress-induced apoptosis through its ability to induce the upregulation of DR5 (death Receptor 5), which in turn activates caspase 8 and BAX [21]. Furthermore, others have shown that the upregulation of BIM induced by CHOP plays a central role in ER stress-triggered apoptosis, as well as downregulation of BCL-2 [28,29,30]. We therefore tested the expression of BCL-2 and BIM in the Ramos and BL2 cells before and after treatment with VT-1. BCL-2 was expressed in BL2 cells, but its level was not downregulated by VT-1 treatment (even slightly increased), whereas this protein was not present in Ramos cells (Figure 5). By contrast, BIM was not expressed in BL2 cells but was present in Ramos cells. However, VT-1 treatment clearly induced a decrease in the level of BIM in these cells. These results show that the role of CHOP in VT-1 induced apoptosis is not through BCL-2 or BIM regulation. 

**Figure 6:**
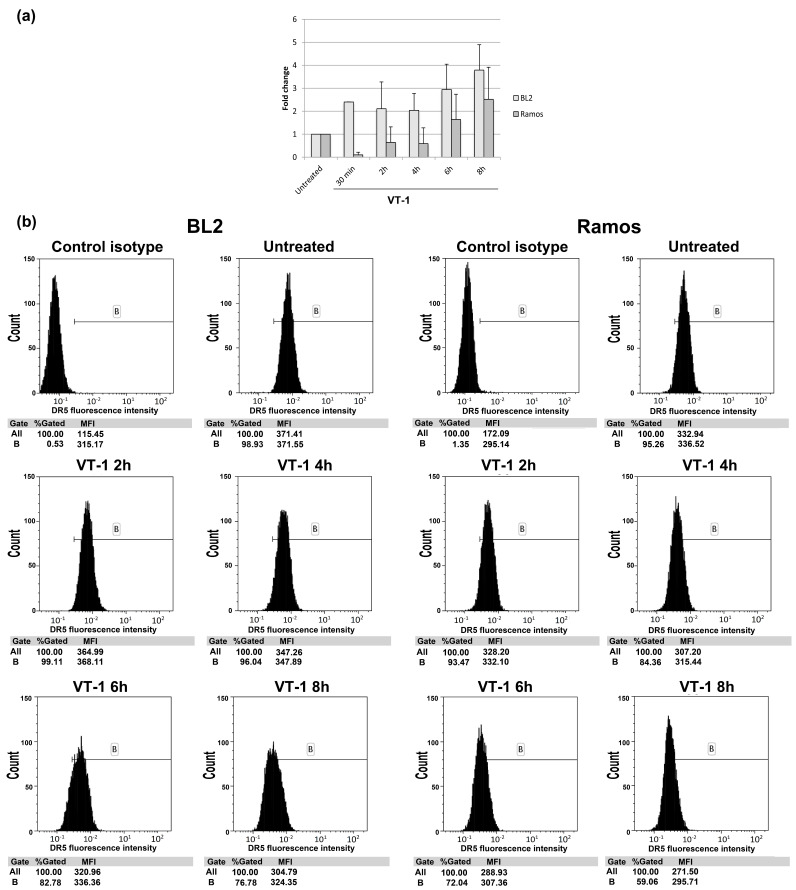
VT-1 treatment does not increase the expression of the DR5 receptor at the surface of BL cells. BL2 and Ramos cells were treated with VT-1 for 0, 2, 4, 6, or 8 h and then DR5 expression analyzed by qRT-PCR (**a**) and flow cytometry (**b**).

We then assessed DR5 expression in the BL2 and Ramos cell lines after VT-1 treatment. DR5 mRNA levels increased in the two cell lines after VT-1 treatment (Figure 6a). However, this result was not confirmed by the analysis of DR5 expression at the cell surface by flow cytometry. Indeed, up to 100% of the cells clearly expressed DR5 before treatment and its expression gradually decreased between 4 and 8 h of VT-1 treatment, as shown by the mean fluorescence intensity (MFI) reported under each graph in Figure 6b (MFI decrease from 371 to 324 for BL2 cells and from 336 to 295 for Ramos cells). Overall, our results suggest that in certain BL cells, CHOP participates in VT-1-induced apoptotic signaling but not through already-known mechanisms involving the BCL-2 family or DR5. 

### 2.4. Calpain Activation Is Not Involved in VT-1-Induced Apoptosis

**Figure 7:**
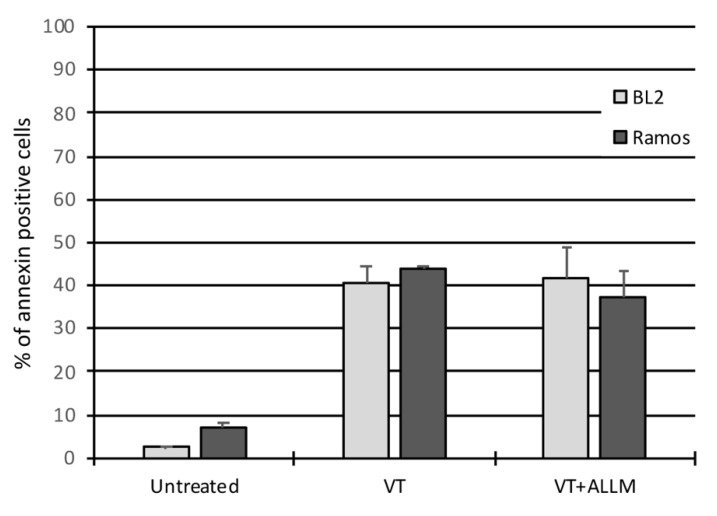
VT-1-induced apoptosis is independent of calpain activation. Cells were pretreated with ALLM (calpain and cathepsin inhibitor) for 30 min and then VT-1. After 6 h of treatment, cells were analyzed by flow cytometry using annexin V/PI.

We further investigated the role of ER stress in VT-1-induced apoptosis by assessing the role of calcium in this pathway. Indeed, the activation of ER stress is frequently accompanied by calcium release into the cytosol and an increase in the calcium concentration has also been implicated in the induction of apoptosis [31,32]. Notably, Calpain, a cysteine protease activated by elevated intracellular Ca^2+^ levels induced by ER stress can act as an alternative pathway to activate caspases. We determined whether calpain activation is involved in VT-1-induced apoptosis by pretreating cells with ALLM (calpain and cathepsin inhibitor) for 30 min and then induced apoptosis with VT-1. After 6 h of treatment, the cells were analyzed by flow cytometry after annexin V/PI labeling. ALLM had no effect on VT-1-induced apoptosis either in BL2 nor in Ramos cells (Figure 7). These results suggest that the induction of apoptosis by VT-1 in BL cells relies on a Ca^2+^-independent signaling pathway.

## 3. Discussion

**Figure 8:**
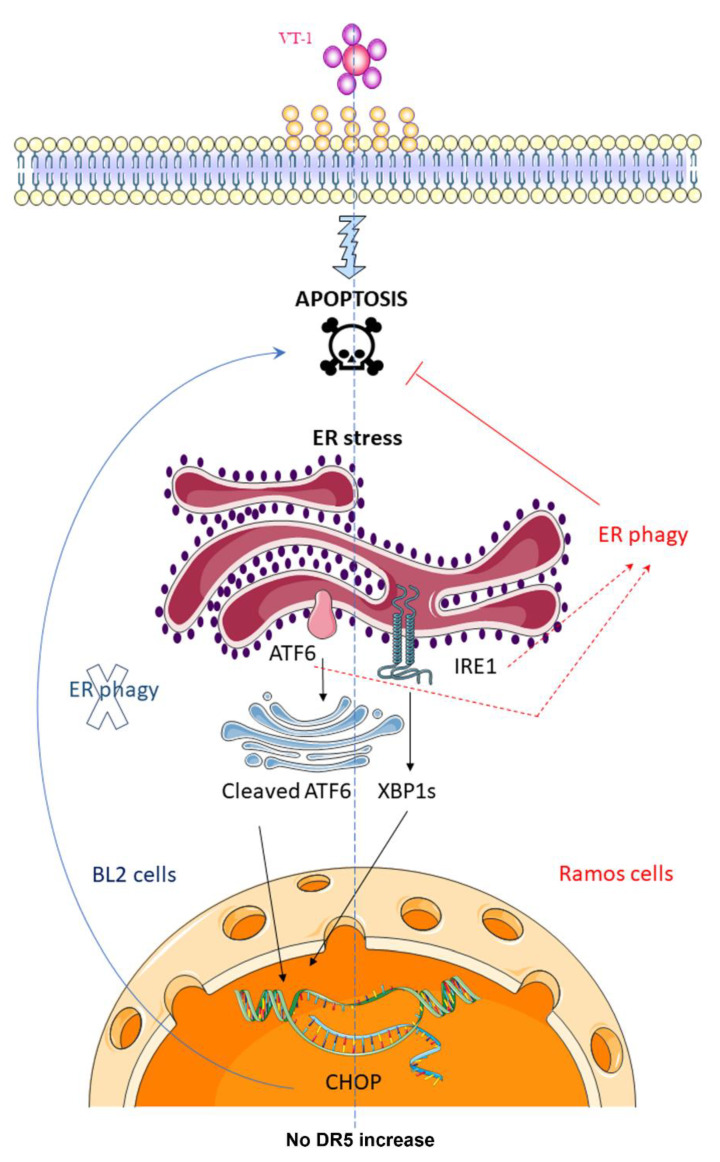
VT-1-induced ER stress triggers apoptotic or survival pathways in Burkitt lymphoma cells. We showed previously that the signal transduction pathway induced by VT-1 in BL cells is a relatively conventional caspase- and mitochondria-dependent pathway [17,18,19]. Here, we report that treating cells with VT-1 induces the ER stress response by activation of the ER stress sensors IRE1 and ATF6, followed by increased expression of the transcription factor CHOP. Interestingly, we observed differential roles for CHOP, which appears to be part of the VT-1-induced apoptotic pathway in BL2 cells, whereas it is not implicated in the death process of Ramos cells. On the contrary, ER-phagy which occurred in these cells restrained VT-1-induced apoptosis. The role of CHOP in Ramos cells and the pathway (ATF6 or/and IRE1) involved in triggering ER-phagy are still not known. Dashed lines indicate possible mechanisms, solid lines indicate demonstrated effects.

Numerous reports have shown that VT-1 is able to trigger multiple effector pathways that, for the vast majority, lead to apoptosis of the cells [13,14,15]. Here, we found that treating BL cells with VT-1 induced the activation of IRE1 and ATF6, the two main sensors of ER stress that operate at the transcriptional level, but not the third one, PERK, which is directly involved in globally shutting off mRNA translation. We also showed that VT-1 treatment results in the upregulation of CHOP, which is normally the point at which ER stress pathways switch from the restoration of homeostasis to programmed cell death. However, we observed differential roles for CHOP, which appears to be part of the VT-1-induced apoptotic pathway in BL2 cells, whereas it is not implicated in the death process of Ramos cells. On the contrary, a selective autophagy pathway called ER-phagy restrained VT-1-induced apoptosis in these cells (Figure 8). It is possible that ER-phagy plays a protective role by preventing the toxic accumulation of unfolded proteins in the ER resulting from the VT-1-induced inhibition of protein synthesis or by altering intracellular toxin routing (and promoting its proteolytic degradation). 

UPR, the cellular response to ER stress, is intrinsically related to autophagy, which acts as a cytoprotective factor and proceeds through two interconnected pathways, ER stress-mediated autophagy and ER-phagy. ER-phagy is a recently identified form of selective autophagy and there are still many questions about its molecular mechanisms and physiological role. However, it is generally accepted that the core autophagy machinery is required for its activation [33]. Others have studied the role of autophagy in Stxs-induced apoptosis [34,35,36] and all have shown that autophagy inhibitors protect against toxin cytotoxicity, suggesting that autophagy participates in the cell death mechanism. Interestingly, Lee et al. showed that autophagy is induced by Stxs, both in toxin-sensitive and toxin-resistant cells, but that calpains and caspases can cleave ATG5 and BECLIN only in sensitive cells and thus transform a pro-survival autophagic response to an apoptotic response. The discrepancies between these results and ours concerning the role of autophagy in VT/Stx-induced apoptosis may be due to the different cell types studied. 

In neuroblastoma cells, Ogata et al. showed that the ER stressors thapsigargin and tunicamycin induce the formation of autophagosomes via the IRE1/JNK pathway, whereas PERK and ATF6 appear not to be involved [37], and that such IRE1/JNK-induced autophagy protects the cells against death induced by ER stress. In this context, it is interesting to note that starvation-induced autophagy is mediated by the specific phosphorylation of ER-localized BCL-2 by JNK1, which leads to disruption of the BCL2/BECLIN complex [38]. On the other hand, others have shown that phosphorylation of BCL-2 can disrupt the association of BCL-2 either with BECLIN or BAX, thus contributing to both autophagy and apoptosis, and that these events can occur sequentially in the same cell [39]. Finally, it has also been shown that the IRE1/XBP-1s axis induces autophagy, with increased conversion of LC3I to LC3II and increased expression of BECLIN [40]. In our BL model, we show that VT-1 induces the ER stress response through the activation of ATF6 and IRE1/XBP-1s, suggesting that one of these two arms is responsible for the induction of ER-phagy. However, there was no activation or involvement of calpain, no increased expression or cleavage of BECLIN, and no change in BCL-2 phosphorylation (data not show) in our cell lines after VT-1 treatment, consistent with a pro-survival effect of autophagy but not through activation of the IRE1/JNK/XBP-1s pathways. 

ATF6 has also been shown to contribute to ER expansion [41] and autophagy, notably through its interaction with C/EBP-β and the ability of this complex to induce the expression of DAPK1 [42], a kinase that can phosphorylate BECLIN, thus releasing it from BCL-XL and resulting in autophagosome formation [43]. In our model, we were unable to detect a change in BECLIN phosphorylation (data not shown) after VT-1 treatment, which does not favor a role for DAPK1. ATF6 may also be involved in the initiation of autophagy by upregulating the expression of BIP [44]. Indeed, BIP has been shown to modulate AKT signaling, which is a well-known regulator of mTOR-mediated autophagy [45]. Since the IRE1 arm does not appear to be involved in the induction of ER-phagy in our BL model, it would certainly be worthwhile to investigate the role of ATF6 in this process. 

We show that CHOP is upregulated in BL2 cells during VT-1 treatment and that it participates in the induction of the apoptotic pathway. However, we also show that the effect of CHOP on apoptosis is not due—contrary to what has been previously reported [21]—to the downregulation of BCL-2 and upregulation of DR5. This raises the question of whether CHOP is directly involved in the apoptotic pathways or whether it inhibits the protection conferred by ER-phagy. In our case, it is possible that CHOP induced a pathway that inhibited ER-phagy in BL2 cells but that this pathway was not activated in Ramos cells. Others have suggested that CHOP may promote ER stress-induced apoptosis via the inhibition of autophagy. They showed that specific shRNA inhibition of CHOP in hepatocellular carcinoma resulted in enhanced tunicamycin-induced autophagy (shown by increased LC3 II expression) and reduced apoptosis [46]. On the other hand, it has been shown that CHOP can directly promote PUMA (p53 upregulated modulator of apoptosis) expression in certain circumstances of induced ER stress and that the CHOP/PUMA axis can synergize with the classical apoptotic process [47]. The role of CHOP in VT-1-induced apoptosis of BL cells is yet to be elucidated. 

We previously reported that treatment of BL cell lines with VT-1 induces a caspase- and mitochondria-dependent apoptotic pathway in which BID is essential [17,18,19]. Here, VT-1 also simultaneously induced ER stress, which activated different signaling pathways, depending on the cell line. The kinetics of ER stress induction (between 2 and 4 h) after VT-1 treatment would be compatible with an effect on caspase 8 activation, as previously reported in other models [48]. However, we previously showed that caspase 8 is most likely controlled by c-FLIP_L_ degradation, which occurs via the ubiquitin–proteasome pathway [17]. Furthermore, the fact that ER stress contributed to either survival or death in different BL cell lines suggests that it is not necessary for VT-1 induced apoptosis. 

Because Gb3 functions as a receptor for Stxs and its expression is deregulated in various malignancies [8,9,10], several engineered Stxs are currently under investigation as potential anti-cancer agents. However, the utility of Stxs is considered to be limited, as it induces endothelial cell damage and is responsible for HUS observed in patients infected with Shiga toxin-producing *Escherichia coli* [12]. Furthermore, another detrimental effect is that Stxs can damage human hematopoietic progenitor cells since they expressed Gb3 and Gb4 receptors at the primary stage of erythropoietic differentiation [49]. A better understanding of the molecular mechanisms that determine the outcome of Stxs-induced ER stress and autophagy in each cell type will certainly offer new opportunities to improve their potential as cancer therapies. Indeed, it is now clear that, depending on the cell type, Stxs can induce different signaling pathways, such as autophagy and ER stress, which activate both pro-survival and pro-death mechanism and thereby play a dual role in VT-1-mediated killing. It is thus possible that stimulating or blocking certain pathways could improve the specific action of the toxin, depending on the cell type (normal versus tumor). Future investigations will be necessary to more precisely determine whether combining VT-1 with molecules that enhance autophagy could protect normal cells while killing tumor cells. 

## 4. Materials and Methods 

### 4.1. Cell Lines

The Ramos cell line was obtained from the American type culture collection (ATCC-CRL-1596, Rockville, MD, USA). The BL2 cell line was originally established from a case of BL and kindly provided by the International Agency for Research on Cancer (IARC, Lyon, France). Both cell lines were previously tested for Gb3 expression and VT-1 sensitivity [15,17,18,19]. Mission® shRNA lentiviral transduction particles (Sigma Aldrich, Saint-Quentin Fallavier, France) were used to suppress CHOP gene expression (SHCLNV-TRCN00000007263, 7264) and Mission® non-target shRNA control lentiviral transduction particles (Sigma Aldrich, Saint-Quentin Fallavier, France) were used as a control (SHC002V). The 2 × 10^6^ BL cells were transduced with lentiviral particles (multiplicity of infection (MOI) of 15) in fresh media distributed in 6 wells plates. Cells were then incubated for 24 h at 37 °C in a humidified incubator in an atmosphere of 5% CO_2_. The media containing lentiviral particles was then replaced by fresh media and incubation was continued for 24 h. Cells were then cultured in media supplemented with selection agent (0.6 μg/mL puromycin) for several weeks. Two CHOP-repressed cell lines were established for each parental cell line: Ramos shCHOP1, Ramos shCHOP2 and BL2 shCHOP1, BL2 shCHOP2. Two control cell lines: Ramos shCTRL and BL2 shCTRL (which behave like the parental Ramos and BL2 cell lines) were also established.

All cell lines were cultured in RPMI 1640 medium (Life technology, Villebon sur Yvette, France) containing 2 mM l-glutamine, 1 mM sodium pyruvate, 20 mM glucose, 100 U/mL penicillin, and 100 μg/mL streptomycin and supplemented with 7% heat-inactivated fetal calf serum.

### 4.2. Total Extract Preparation

Aliquots of 1 × 10^6^ cells were pelleted, solubilized in ice-cold RIPA buffer (150 mM NaCl, 50 mM Tris, pH 7.5, 5 mM EDTA, 0.5% NP40, 0.5% NaDOC, 0.1% SDS, complete protease inhibitor) and sonicated. Then the samples were analyzed by western blotting.

### 4.3. Western-Blot Analysis

The protocol for western-blotting was described elsewhere [17]. Antibodies used: ATF6 (IMGENEX, clone 70B1413.1, Noyal Chatillon sur Seiche, France), Phospho- eIF2α (Abcam, clone E90, Cambridge, UK), eIF2α (Santa Cruz, clone D-3, Heidelberg, Germany), CHOP (Santa Cruz, clone B-3), and ATF4 (Santa Cruz, clone c-20).

### 4.4. Detection of XBP-1 mRNA Splicing

After total RNA extraction, RT-PCR products of XBP-1 mRNA were obtained using primers 5′-cggtgcgcggtgcgtagtctgga-3′ (sense) and 5′-tgaggggctgagaggtgcttcct-3′ (anti-sense). Upon activation of XBP-1 mRNA, a 26 bp fragment containing an Apa-LI site is spliced. Therefore, the RT-PCR products were digested with Apa-LI to distinguish the spliced form from the unspliced form. The inactive form was revealed by electrophoresis as two cleaved fragments and the active form as a non-cleaved fragment.

### 4.5. Analysis of DR5 Cell-Surface Expression

BL cells were incubated with or without VT-1 (5 ng/mL) for 0, 2, 4, 6, or 8 h. Cells were then incubated with antibodies directed against membrane-bound DR5 (Santa Cruz, clone DJR2-4) for at least 30 min at 4 °C. Following incubation, cells were washed and then incubated with secondary antibody (goat anti-mouse Alexa 488, Life technologies) for at least 30 min at 4 °C. Finally, cells were washed and suspended in 0.2 mL PBS. Cell fluorescence was measured by flow cytometry (Accuri C6 cytometer, Becton-Dickinson, Pont-de-Claix, France). Untreated cells and cells incubated with the isotype control and then the fluorescein-labeled secondary antibody served as controls.

### 4.6. Cell Death Measurement

Cell death was assessed by labeling cells with annexin V-FITC and propidium iodide (PI). Aliquots of 1 × 10^6^ cells were incubated for 6 h at 37 °C with VT-1 (5 ng/mL) in 1 mL of complete RPMI medium. The staining protocol was described previously [17] 

### 4.7. Electron Microscopy

Cells were fixed in 2% glutaraldehyde in 0.1 M cacodylate buffer, pH 7.4 for 1 h at room temperature. They were then post-fixed for 1 h in 1% osmium tetroxide mixed with 1.5% potassium ferrocyanide in the same buffer. The samples were then exposed to successive baths of increasing ethanol concentration for dehydration and embedded in epoxy resin (Embed 812, EMS 14120). Once the resin had cured, ultra-thin sections (70 nm) were performed and collected on copper grids coated with collodion-carbon (EMS, G200-Cu). They were stained with a 2% aqueous solution of uranyl acetate, followed by a staining with lead citrate in Reynold’s solution. Finally, the sections were observed with the Zeiss 902 electron microscope (Carl Zeiss microscopy GmbH, Jena, Germany), using the Megaview III CCD camera (Olympus Soft Imaging Solutions, GmbH, Münster, Germany).

### 4.8. Data Analysis and Statistics

All values for statistical significance represent the mean ± standard deviation (s.d.). Statistical analyses were performed using the non-parametric Mann Whitney test for side-by-side comparisons. Differences were considered to be statistically significance for *p* < 0.05. 

## References

[1] Boyer O., Niaudet P. (2011). Hemolytic uremic syndrome: New developments in pathogenesis and treatment. Int. J. Nephrol..

[2] Joseph A., Cointe A., Mariani Kurkdjian P., Rafat C., Hertig A. (2020). Shiga Toxin-Associated Hemolytic Uremic Syndrome: A Narrative Review. Toxins.

[3] Jacewicz M., Clausen H., Nudelman E., Donohue-Rolfe A., Keusch G.T. (1986). Pathogenesis of shigella diarrhea. XI. Isolation of a shigella toxin-binding glycolipid from rabbit jejunum and HeLa cells and its identification as globotriaosylceramide. J. Exp. Med..

[4] DeGrandis S., Law H., Brunton J., Gyles C., Lingwood C.A. (1989). Globotetraosylceramide is recognized by the pig edema disease toxin. J. Biol. Chem..

[5] Johannes L. (2017). Shiga Toxin-A Model for Glycolipid-Dependent and Lectin-Driven Endocytosis. Toxins.

[6] Bergan J., Dyve Lingelem A.B., Simm R., Skotland T., Sandvig K. (2012). Shiga toxins. Toxicon.

[7] Nudelman E., Kannagi R., Hakomori S., Parsons M., Lipinski M., Wiels J., Fellous M., Tursz T. (1983). A glycolipid antigen associated with Burkitt lymphoma defined by a monoclonal antibody. Science.

[8] Arab S., Russel E., Chapman W.B., Rosen B., Lingwood C.A. (1997). Expression of the verotoxin receptor glycolipid, globotriaosylceramide, in ovarian hyperplasias. Oncol Res..

[9] Gupta V., Bhinge K.N., Hosain S.B., Xiong K., Gu X., Shi R., Ho M.Y., Khoo K.H., Li S.C., Li Y.T. (2012). Ceramide glycosylation by glucosylceramide synthase selectively maintains the properties of breast cancer stem cells. J. Biol. Chem..

[10] Ohyama C., Fukushi Y., Satoh M., Saitoh S., Orikasa S., Nudelman E., Straud M., Hakomori S. (1990). Changes in glycolipid expression in human testicular tumor. Int. J. Cancer.

[11] Bien T., Perl M., Machmüller A.C., Nitsche U., Conrad A., Johannes L., Müthing J., Soltwisch J., Janßen K.P., Dreisewerd K. (2020). MALDI-2 Mass Spectrometry and Immunohistochemistry Imaging of Gb3Cer, Gb4Cer, and Further Glycosphingolipids in Human Colorectal Cancer Tissue. Anal. Chem..

[12] Engedal N., Skotland T., Torgersen M.L., Sandvig K. (2011). Shiga toxin and its use in targeted cancer therapy and imaging. Microb. Biotechnol..

[13] Lee S.Y., Cherla R.P., Caliskan I., Tesh V.L. (2005). Shiga toxin 1 induces apoptosis in the human myelogenous leukemia cell line THP-1 by a caspase-8-dependent, tumor necrosis factor receptor-independent mechanism. Infect. Immun..

[14] Ching J.C., Jones N.L., Ceponis P.J., Karmali M.A., Sherman P.M. (2002). Escherichia coli shiga-like toxins induce apoptosis and cleavage of poly(ADP-ribose) polymerase via in vitro activation of caspases. Infect. Immun..

[15] Mangeney M., Lingwood C.A., Taga S., Caillou B., Tursz T., Wiels J. (1993). Apoptosis induced in Burkitt’s lymphoma cells via Gb3/CD77, a glycolipid antigen. Cancer Res..

[16] Fujii J., Matsui T., Heatherly D.P., Schlegel K.H., Lobo P.I., Yutsudo T., Ciraolo G.M., Morris R.E., Obrig T. (2003). Rapid apoptosis induced by Shiga toxin in HeLa cells. Infect. Immun..

[17] Garibal J., Hollville E., Renouf B., Tetaud C., Wiels J. (2010). Caspase-8-mediated cleavage of Bid and protein phosphatase 2A-mediated activation of Bax are necessary for Verotoxin-1-induced apoptosis in Burkitt’s lymphoma cells. Cell Signal..

[18] Tetaud C., Falguieres T., Carlier K., Lecluse Y., Garibal J., Coulaud D., Busson P., Steffensen R., Clausen H., Johannes L. (2003). Two distinct Gb3/CD77 signaling pathways leading to apoptosis are triggered by anti-Gb3/CD77 mAb and verotoxin-1. J. Biol. Chem..

[19] Debernardi J., Hollville E., Lipinski M., Wiels J., Robert A. (2018). Differential role of FL-BID and t-BID during verotoxin-1-induced apoptosis in Burkitt’s lymphoma cells. Oncogene.

[20] Bertolotti A., Zhang Y., Hendershot L.M., Harding H.P., Ron D. (2000). Dynamic interaction of BiP and ER stress transducers in the unfolded-protein response. Nat. Cell Biol..

[21] Lee S.Y., Lee M.S., Cherla R.P., Tesh V.L. (2008). Shiga toxin 1 induces apoptosis through the endoplasmic reticulum stress response in human monocytic cells. Cell Microbiol..

[22] Lee M.S., Cherla R.P., Lentz E.K., Leyva-Illades D., Tesh V.L. (2010). Signaling through C/EBP homologous protein and death receptor 5 and calpain activation differentially regulate THP-1 cell maturation-dependent apoptosis induced by Shiga toxin type 1. Infect. Immun..

[23] Fujii J., Wood K., Matsuda F., Carneiro-Filho B.A., Schlegel K.H., Yutsudo T., Binnington-Boyd B., Lingwood C.A., Obata F., Kim K.S. (2008). Shiga toxin 2 causes apoptosis in human brain microvascular endothelial cells via C/EBP homologous protein. Infect. Immun..

[24] Hetz C. (2012). The unfolded protein response: controlling cell fate decisions under ER stress and beyond. Nat. Rev. Mol. Cell Biol..

[25] Haze K., Yoshida H., Yanagi H., Yura T., Mori K. (1999). Mammalian transcription factor ATF6 is synthesized as a transmembrane protein and activated by proteolysis in response to endoplasmic reticulum stress. Mol. Biol. Cell.

[26] Harding H.P., Zhang Y., Bertolotti A., Zeng H., Ron D. (2000). Perk is essential for translational regulation and cell survival during the unfolded protein response. Mol. Cell.

[27] Yoshida H., Matsui T., Yamamoto A., Okada T., Mori K. (2001). XBP1 mRNA is induced by ATF6 and spliced by IRE1 in response to ER stress to produce a highly active transcription factor. Cell.

[28] McCullough K.D., Martindale J.L., Klotz L.O., Aw T.Y., Holbrook N.J. (2001). Gadd153 sensitizes cells to endoplasmic reticulum stress by down-regulating Bcl2 and perturbing the cellular redox state. Mol. Cell Biol..

[29] Puthalakath H., O’Reilly L.A., Gunn P., Lee L., Kelly P.N., Huntington N.D., Hughes P.D., Michalak E.M., McKimm-Breschkin J., Motoyama N. (2007). ER stress triggers apoptosis by activating BH3-only protein Bim. Cell.

[30] Yamaguchi H., Wang H.G. (2004). CHOP is involved in endoplasmic reticulum stress-induced apoptosis by enhancing DR5 expression in human carcinoma cells. J. Biol. Chem..

[31] Carreras-Sureda A., Pihán P., Hetz C. (2018). Calcium signaling at the endoplasmic reticulum: fine-tuning stress responses. Cell Calcium.

[32] Orrenius S., Zhivotovsky B., Nicotera P. (2003). Regulation of cell death: the calcium-apoptosis link. Nat. Rev. Mol. Cell Biol..

[33] Song S., Tan J., Miao Y., Zhang Q. (2018). Crosstalk of ER stress-mediated autophagy and ER-phagy: Involvement of UPR and the core autophagy machinery. J. Cell Physiol..

[34] Lee M.S., Cherla R.P., Jenson M.H., Leyva-Illades D., Martinez-Moczygemba M., Tesh V.L. (2011). Shiga toxins induce autophagy leading to differential signalling pathways in toxin-sensitive and toxin-resistant human cells. Cell Microbiol..

[35] Sandvig K., Garred O., Prydz K., Kozlov J.V., Hansen S.H., van Deurs B. (1992). Retrograde transport of endocytosed Shiga toxin to the endoplasmic reticulum. Nature.

[36] Tang B., Li Q., Zhao X.H., Wang H.G., Li N., Fang Y., Wang K., Jia Y.P., Zhu P., Gu J. (2015). Shiga toxins induce autophagic cell death in intestinal epithelial cells via the endoplasmic reticulum stress pathway. Autophagy.

[37] Ogata M., Hino S., Saito A., Morikawa K., Kondo S., Kanemoto S., Murakami T., Taniguchi M., Tanii I., Yoshinaga K. (2006). Autophagy is activated for cell survival after endoplasmic reticulum stress. Mol. Cell Biol..

[38] Wei Y., Pattingre S., Sinha S., Bassik M., Levine B. (2008). JNK1-mediated phosphorylation of Bcl-2 regulates starvation-induced autophagy. Mol. Cell.

[39] Wei Y., Sinha S., Levine B. (2008). Dual role of JNK1-mediated phosphorylation of Bcl-2 in autophagy and apoptosis regulation. Autophagy.

[40] Margariti A., Li H., Chen T., Martin D., Vizcay-Barrena G., Alam S., Karamariti E., Xiao Q., Zampetaki A., Zhang Z. (2013). XBP1 mRNA splicing triggers an autophagic response in endothelial cells through BECLIN-1 transcriptional activation. J. Biol. Chem..

[41] Bommiasamy H., Back S.H., Fagone P., Lee K., Meshinchi S., Vink E., Sriburi R., Frank M., Jackowski S., Kaufman R.J. (2009). ATF6alpha induces XBP1-independent expansion of the endoplasmic reticulum. J. Cell Sci..

[42] Gade P., Ramachandran G., Maachani U.B., Rizzo M.A., Okada T., Prywes R., Cross A.S., Mori K., Kalvakolanu D.V. (2012). An IFN-gamma-stimulated ATF6-C/EBP-beta-signaling pathway critical for the expression of Death Associated Protein Kinase 1 and induction of autophagy. Proc. Natl. Acad. Sci. USA.

[43] Zalckvar E., Berissi H., Mizrachy L., Idelchuk Y., Koren I., Eisenstein M., Sabanay H., Pinkas-Kramarski R., Kimchi A. (2009). DAP-kinase-mediated phosphorylation on the BH3 domain of beclin 1 promotes dissociation of beclin 1 from Bcl-XL and induction of autophagy. EMBO Rep..

[44] Yoshida H., Haze K., Yanagi H., Yura T., Mori K. (1998). Identification of the cis-acting endoplasmic reticulum stress response element responsible for transcriptional induction of mammalian glucose-regulated proteins. Involvement of basic leucine zipper transcription factors. J. Biol. Chem..

[45] Yung H.W., Charnock-Jones D.S., Burton G.J. (2011). Regulation of AKT phosphorylation at Ser473 and Thr308 by endoplasmic reticulum stress modulates substrate specificity in a severity dependent manner. PLoS ONE.

[46] Lei Y., Wang S., Ren B., Wang J., Chen J., Lu J., Zhan S., Fu Y., Huang L., Tan J. (2017). CHOP favors endoplasmic reticulum stress-induced apoptosis in hepatocellular carcinoma cells via inhibition of autophagy. PLoS ONE.

[47] Lee Y.S., Lee D.H., Choudry H.A., Bartlett D.L., Lee Y.J. (2018). Ferroptosis-Induced Endoplasmic Reticulum Stress: Cross-talk between Ferroptosis and Apoptosis. Mol. Cancer Res..

[48] Iurlaro R., Muñoz-Pinedo C. (2016). Cell death induced by endoplasmic reticulum stress. FEBS J..

[49] Betz J., Dorn I., Kouzel I.U., Bauwens A., Meisen I., Kemper B., Bielaszewska M., Mormann M., Weymann L., Sibrowski W. (2016). Shiga toxin of enterohaemorrhagic Escherichia coli directly injures developing human erythrocytes. Cell Microbiol..

